# SIRT1 Activating compounds reduce oxidative stress mediated neuronal loss in viral induced CNS demyelinating disease

**DOI:** 10.1186/2051-5960-2-3

**Published:** 2014-01-02

**Authors:** Reas S Khan, Kimberly Dine, Jayasri Das Sarma, Kenneth S Shindler

**Affiliations:** 1 Department of Ophthalmology, Scheie Eye Institute and FM Kirby Center for Molecular Ophthalmology, Stellar-Chance Laboratories, University of Pennsylvania, Philadelphia, PA 19104, USA; 2 Department of Biological Science, Indian Institute of Science Education and Research-Kolkata (IISER-K), Mohanpur Campus Mohanpur, Nadia, West Bengal 741252, India

**Keywords:** Demyelinating disease, Mouse hepatitis virus, SIRT1, Neuroprotection, Oxidative stress, Optic neuropathy

## Abstract

**Background:**

Multiple sclerosis (MS) is characterized by central nervous system inflammation and demyelination, and increasing evidence demonstrates significant neuronal damage also occurs and is associated with permanent functional impairment. Current MS therapies have limited ability to prevent neuronal damage, suggesting additional neuroprotective therapies are needed. Compounds that activate the NAD^+^-dependent SIRT1 deacetylase prevent neuronal loss in an autoimmune-mediated MS model, but the mechanism of this effect is unknown, and it is unclear whether SIRT1 activating compounds exert similar effects in demyelinating disease induced by other etiologies. We measured neuronal loss in C57BL/6 mice inoculated with a neurotropic strain of mouse hepatitis virus, MHV-A59, that induces an MS-like disease.

**Results:**

Oral treatment with the SIRT1 activating compound SRTAW04 significantly increased SIRT1 activity within optic nerves and prevented neuronal loss during optic neuritis, an inflammatory demyelinating optic nerve lesion that occurs in MS and its animal models. MHV-A59 induced neuronal loss was associated with reactive oxygen species (ROS) accumulation, and SRTAW04 treatment significantly reduced ROS levels while promoting increased expression of enzymes involved in mitochondrial function and reduction of ROS. SRTAW04 exerted similar protective effects in EAE spinal cords, with decreased demyelination.

**Conclusions:**

Results demonstrate that SIRT1 activating compounds prevent neuronal loss in viral-induced demyelinating disease similar to their effects in autoimmune-mediated disease. One mechanism of this neuroprotective effect involves increasing mitochondrial biogenesis with reduction of oxidative stress. SIRT1 activators represent a potential neuroprotective therapy for MS. Understanding common mechanisms of these effects in distinct disease models will help identify targets for more specific therapies.

## Background

Multiple sclerosis (MS) is an inflammatory demyelinating disease of the central nervous system (CNS) [1]. Significant neuronal damage also occurs in MS, and correlates with permanent neurologic dysfunction [2-6]. Current therapies reduce the inflammatory component of MS, but their ability to prevent neuronal damage is limited [7-10], suggesting additional therapies with neuroprotective benefits are needed.

While evidence suggests MS is an autoimmune disease against CNS myelin, the exact etiology is not known. Other evidence suggests genetic and viral-mediated triggers [11]. Because the etiology is unknown, several MS animal models are used. The most common MS model, experimental autoimmune encephalomyelitis (EAE), is an autoimmune driven CNS demyelinating disease [12] that also exhibits neuronal damage [13-15]. Another MS model is induced by infection with a neurotropic strain of mouse hepatitis virus (MHV), MHV-A59 [16,17]. While MHV-A59 induces CNS inflammation and demyelination similar to EAE, the etiology is distinct, involving direct neuronal infection, and the demyelination is not dependant on an intact immune system [18]. Therefore, these models provide unique and contrasting systems for studying potential neuroprotective strategies for MS.

Optic nerve is a frequent site of MS lesions, and optic neuritis is a common presenting sign of MS [19]. Similarly, optic neuritis occurs at high frequency in both EAE and MHV models of MS [20-22]. In addition to optic nerve inflammation, these studies demonstrate significant axonal damage, with loss of retinal ganglion cell (RGC) neurons that comprise the optic nerve also found in EAE optic neuritis, but not examined in the MHV model. RGCs are readily quantified, allowing optic neuritis to serve as a representative lesion for assessing neuronal damage [5,6,14,20,22].

Activation of SIRT1, an NAD-dependent deacetylase involved in cell stress responses, attenuates neuronal damage in EAE [23-25], although mechanisms mediating this effect are not known. SIRT1 reduces oxidative stress and promotes mitochondrial biogenesis in muscle [26] and cultures of neuronal cells [27], and oxidative stress plays a role in neuronal degeneration in MS and EAE [28-30], suggesting SIRT1 activation may prevent neuronal damage by increasing mitochondrial function and reducing oxidative stress.

While mechanisms of neuronal damage in MHV-A59-induced demyelinating disease are not fully understood, oxidative stress is a common mechanism of cellular injury and is likely to occur in MHV-A59 infection. The current studies characterize mechanisms of neuronal loss in viral-induced demyelinating disease, and examine the ability of SIRT1 activating compounds to prevent neuronal loss, reduce oxidative stress, and regulate proteins that promote mitochondrial function.

## Methods

### Mice

Four-week-old female C57BL/6 (B6) mice were purchased from the Jackson Laboratory (Bar Harbor, ME). Treatment of the animals was reviewed and approved by the Institutional Animal Care and Use Committee at the University of the Pennsylvania, where the mice were maintained and fed ad libitum in an approved animal care facility.

#### Inoculation and treatment of mice

MHV-free mice were inoculated intracranially with 50% of the half lethal dose of the demyelinating strain MHV-A59 [2000 plaque forming units (pfu)], a related non-demyelinating strain MHV2 (100 pfu) used as a negative control, or isogenic recombinant strains containing a fluorescent tag: RSA59 (20,000 pfu) used as a positive control and RSMHV2 (250 pfu) as negative control. Due to differences in replication and elimination, these doses result in equivalent viral loads in the CNS, and inoculation of each strain was performed as described previously [31,32]. Mice were monitored daily for mortality and signs of disease [16,17]. Mock-infected controls were inoculated similarly but with an uninfected cell lysate at a comparable dilution. SRTAW04 (Sirtris, a GSK company, Cambridge, MA) was prepared in PBS containing 0.5% methyl cellulose and 0.1% tween 80. Mice were treated with SRTAW04, 100 mg/kg/mouse/day by oral gavage. The dose was chosen based on previously measured pharmacokinetics of the drug and its ability to activate SIRT1 which has been used previously with maximal effects [27,33,34]. Seven day and 30 day treatment groups received SRTAW04 starting from day 1 until sacrifice. To evaluate whether the SRTAW04 target is SIRT1 and its activation, one group of mice received SIRT1 inhibitor EX527 (10 mg/kg/day i.p.) for 30 days [35]. EX527 has the highest inhibitory activity for SIRT1 compared to other analogues and previous studies have shown that EX527 treatment alone does not result in the acetylation of SIRT1/2 target p53 [36,37]. We have previously shown that SIRT1 inhibitors or conditional deletion of the SIRT1 gene, do not exacerbate RGC loss in optic nerve disease models [24,38].

### RGC labeling and counting

RGCs were labeled and counted as described previously [20]. Briefly, 2.5 μL of 1.25% hydroxystilbamidine (Fluorogold; Molecular Probes, Eugene, OR) in sterile water was injected stereotactically into each superior colliculus through holes drilled in the skull. Mice were killed 7 or 30 days after infection and each eye was removed and fixed in 4% paraformaldehyde. Retinas were isolated, prepared as flattened wholemounts, viewed with a fluorescence microscope (Eclipse E600; Nikon, Tokyo, Japan), and photographed at 20X magnification in 12 standard fields: 1/6, 3/6, and 5/6 of the retinal radius from the center of the retina in each quadrant. RGC numbers shown in each experiment represent the total number of RGCs counted per eye (RGCs/0.74 mm2). RGCs were counted by a masked investigator using image analysis software (Image-Pro Plus 5.0; Media Cybernetics, Silver Spring, MD). Alternatively, to confirm RGC numbers, RGC’s were immunolabelled with antibodies to Brn3a. Retinas were dissected and flat as previously reported [39]. The retinas were washed with PBS, permeabilized in 0.5% TritonX 100 in PBS by freezing them for 15 min at −70°C, rinsed in PBS containing 0.5% Triton, and incubated overnight at 4°C with goat-antiBrn3a antibody (Santa Cruz) diluted 1:100 in blocking buffer (PBS, 2% bovine serum albumin, 2% Triton X 100). The retinas were washed three times in PBS, incubated for 2 hours at room temperature (RT) with anti-goat secondary antibody diluted 1:500 in blocking buffer, washed in PBS and mounted vitreous side up on slides in anti-fading solution. Cells were counted in 12 fields as described above for fluorogold labeled RGCs.

### Histopathological analysis

Mice were killed at day 30 post inoculation (p.i.), and were perfused transcardially with PBS followed by PBS containing 4% paraformaldehyde (PFA). Spinal cords or optic nerves were collected, postfixed in 4% PFA overnight and embedded in paraffin. Spinal cord tissues were sectioned at 5 μm and stained with either Luxol Fast Blue (LFB) to detect demyelination or with anti-neurofilament antibodies to detect loss of axons. Areas of demyelination in LFB staining were quantified using a 0–3 point scale as described earlier [21] 0 - no demyelination; 1 - rare foci of demyelination; 2 - a few foci of demyelination; and 3 - large (confluent) areas of demyelination. Two to three sections were examined from each of three spinal cord levels (cervical, thoracic and lumbar) for each mouse. Neurofilament staining was done according to a previously published protocol [40]. Briefly, paraffin embedded sections were dewaxed, rehydrated, and then permeabilized with 0.5% tween-20 in PBS. The sections were blocked with blocking reagent from Vectastain Elite ABC kit and then incubated in the rabbit anti-neurofilament antibody 1:100 (AbCam) at 4°C overnight, washed, and incubated with goat biotinylated anti-rabbit secondary antibody (Invitrogen) for 2 hours at RT. The ABC detection was performed using the Vectastain Elite ABC kit and DAB (diaminobenzidine) substrate kit (both Vector Laboratories, Burlingame, CA) according to manufacturer's instructions. To characterize virus induced inflammation, spinal cords from 7 day p.i. mice were stained with H&E as described previously [21,32] and scored using an inflammation scale: 0 - no inflammation, 0.5 - few inflammatory cells, 1 - inflammatory cells localized near white matter, 1.5 - small patches of inflammatory cells, 2 - large inflammatory plaques, 3 - diffuse inflammation. Experiments were repeated three times with 3–5 mice in each group. All slides were coded and read in a blinded manner. 30 days p.i. spinal cord sections were also stained with a macrophage/microglial marker anti-Iba1 (Wako, Japan) as described previously [18]. Iba1 stained sections were counterstained with hematoxylin (Vector laboratories). Optic nerve tissues were sectioned as 5 μm longitudinal sections and stained with H&E, LFB and anti-neurofilament antibody using methods described above.

### SIRT1 activity assay

SIRT1 activity was determined with a SIRT1 Fluorometric Kit (BIOMOL, Plymouth Meeting, PA) performed according to the manufacturer's instructions. Optic nerves were homogenized in SIRT1 assay buffer, then incubated for 10 min at 37°C to allow degradation of any contaminant NAD^+^. Protein concentration was determined using a BCA protein assay kit (Thermo Scientific, Rockford, IL). Homogenates (20–30 μg protein/well) were then incubated with 100 μM Fluor de Lys–SIRT1 substrate (Enzo Life Sciences) in the presence or absence of NAD^+^ to determine NAD^+^ dependent SIRT1 activity. The reaction was terminated by adding Fluor de Lys Developer (Enzo Life Sciences) and 2 mM nicotinamide after 60 min of incubation at 37°C and fluorescence values were read on a fluorometric plate reader (Wallac Victor^2^ 1420 multi label counter, Perkin Elmer, Waltham, MA) with an excitation wavelength of 360 nm and an emission wavelength of 460 nm. SIRT1 inhibitors nicotinamide (2 mM), suramin (100 μM), and sirtinol (100 μM) were used to confirm the specificity of the reaction.

### MitoSOX staining

MitoSOX Red (Invitrogen) superoxide indicator is a fluorogenic dye for selective detection of superoxide, a reactive oxygen species (ROS), in mitochondria. MitoSOX Red reagent is cell permeable and is selectively targeted to the mitochondria, where it is oxidized by superoxide and exhibits red fluorescence. MitoSOX staining was done according to previously published protocols [38]. Briefly, MitoSOX reagent was diluted to a final concentration of 5 μM in PBS and optic nerves were incubated for 30 min at 37°C. After incubation, nerves were washed three times with PBS, fixed with 4% paraformaldehyde for 10 min and mounted in Optimal Cutting Temperature (OCT) compound (Ted Pella, Inc., Redding, CA). 5 μM cross-sections were made, mounted onto glass slides with Mowiol mounting medium, and observed under an Eclipse E600 (Nikon) fluorescence microscope using excitation 510 nm/emission 580 nm, and photographed at 20X magnification. Photographs were taken centered within each optic nerve cross section by a blinded investigator using a standard exposure, and staining was quantified by calculating the optical density using Image J software (nih.gov).

### Western blot analysis

Optic nerves or retinas in RIPA buffer (150 mM NaCl, 1% NP-40, 0.5% desoxycholic acid, 0.1% SDS and 50 mM Tris, pH 8) were ultrasonicated on ice 5 times for 5 sec each at 10-sec intervals to obtain total protein extracts. Cell lysates were then centrifuged at 14,000 g for 10 min at 4°C, and the protein concentration of the supernatant was determined using a BCA protein assay kit (Thermo Scientific). Sodium dodecyl sulfate polyacrylamide gel electrophoresis was performed on 10% polyacrylamide gels, with 20 μg of protein per lane, and then transferred to nitrocellulose High bound ECL membranes (GE Healthcare Biosciences, Pittsburgh, PA). The membrane was blocked with Odyssey Blocking Buffer (Licor Biotechnology, Lincoln, NE) for 1 hr at RT and probed with rabbit polyclonal antibodies against SIRT1 (1:1500) (Abcam, Cambridge, MA), SOD2 (1:1000) (GeneTex, Irvine, CA) and PGC-1α (1:1000) (Novus Biologicals, Littleton, CO) and mouse monoclonal antibodies against succinate dehydrogenase B (SDHb) (1:1000) (Abcam) overnight at 4°C. After being washed three times using PBS, the membranes were incubated with IRDye® 800CW goat anti-rabbit IgG or IRDye® 600 goat anti-mouse IgG (Licor) as secondary antibodies at a dilution of 1:5000 for 1 hr at RT. After being washed a further three times with PBS, fluorescence was visualized using Odyssey infrared imaging system (Licor). For normalization of signals, blotted membranes were stained for β-actin (Sigma). The intensity of each band was determined using Image J software (nih.gov).

### Statistics

Data are expressed as means ± SEM. Differences in RGC numbers, SIRT1 activity, and protein expression were assessed using one-way ANOVA followed by Student Neuman–Keuls post-hoc test. Statistical differences were considered significant at P < 0.05.

## Results

### MHV-A59 infection induces optic nerve inflammation and neuronal loss

**Figure 1:**
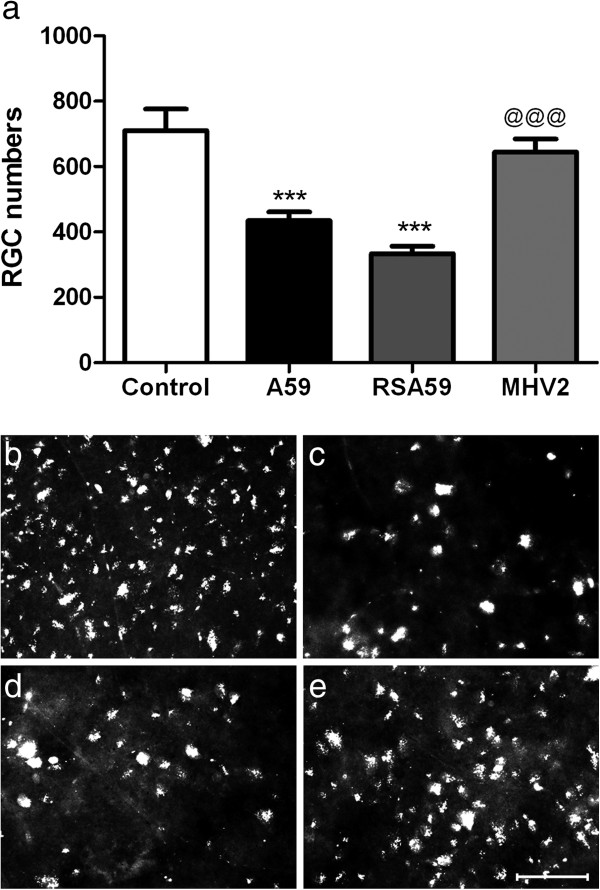
**MHV-A59 infection induces RGC loss. (a)** Inoculation with demyelinating strains MHV-A59 (n = 12), and its isogenic recombinant strain RSA59 (n = 10), lead to significantly decreased (***p < 0.001) RGC numbers compared to non-infected control mice (n = 10), and mice inoculated with the non-demyelinating control strain MHV2 (n = 16) (^@@@^p < 0.001). Mice infected with MHV2 did not show RGC loss compared to non-infected controls. Fluorescently labeled RGCs are shown from a representative field in **(b)** non-infected control and **(e)** MHV2 infected retina, whereas fewer RGCs are seen in corresponding areas of retina in MHV-A59 **(c)** or RSA59 infected mice **(d)**. *Scale bars* 10 μm for **b-e**.

Optic nerves of MHV-A59 infected mice develop inflammation peaking by 5–7 days after inoculation, followed by significant demyelination at day 30; whereas MHV2 infected mice have almost no inflammation nor demyelination [21]. We found similar results in optic nerves stained by H&E and LFB in the current studies (data not shown). To examine whether infection with demyelinating strains of MHV also results in neuronal loss, RGCs were labeled in four-week-old, virus-free, C57BL/6 mice and one week later mice were inoculated with MHV-A59, RSA59 or MHV2. Mice were sacrificed 30 days post-infection and RGCs were counted. Significant differences were found in total RGC numbers counted in 12 standardized retinal fields, but not within any one region of the retina. MHV-A59 infected mice had significantly fewer surviving RGCs compared to non-infected mice (Figure 1). Mice infected with the non-demyelinating control strain MHV2 did not show RGC loss compared to non-infected controls. Consistent with RGC loss induced by MHV-A59, a recombinant strain, RSA59, which is isogenic to MHV-A59 except for an EGFP fluorescent marker and which has similar demyelinating properties [32,41] and ability to induce optic neuritis [42], also induced significant RGC loss compared to non-infected control and MHV2 infected mice (Figure 1).

### SRTAW04 treatment increases SIRT1 activity in optic nerves

**Figure 2:**
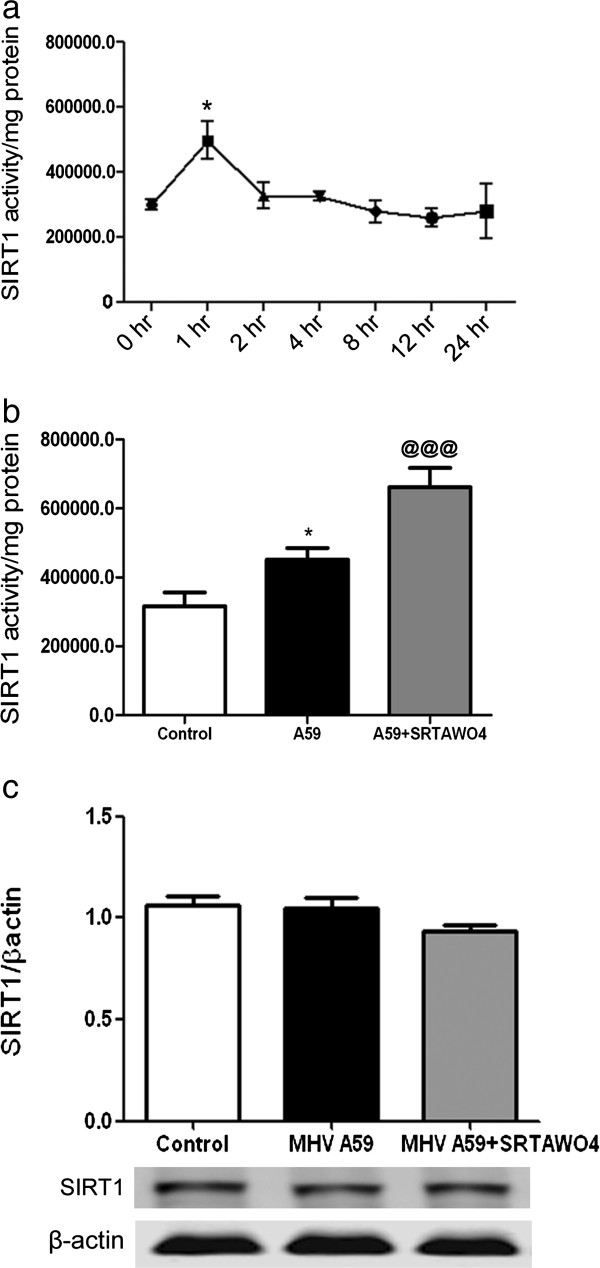
**SRTAW04 treatment increases SIRT1 activity in optic nerves without affecting expression. (a)** Control, MHV-free mice were treated with SIRT1 activator SRTAW04 (100 mg/kg/day) for 4 days and sacrificed on the 4th day at indicated time intervals after the final dose (n = 4 per group). Optic nerves were isolated and SIRT1 activity was determined with a fluorometric substrate assay kit. SIRT1 activity was significantly increased (*p < 0.05) 1 hr after SRTAW04 treatment. Increased activity was transient, returning to control levels after 2 hr. **(b)** SIRT1 activity in the optic nerves of MHV-A59 infected mice after 30 days of SRTAW04 (100 mg/kg/day) treatment (n = 5) showed a significant increase in SIRT1 activity compared to non-infected control (n = 3) (***p < 0.001) and untreated MHV-A59 infected (*p < 0.05) mice (n = 5). **(c)** The expression level of SIRT1 protein in optic nerves of mice after 30 days with or without treatment showed no significant change (n = 4).

SIRT1 activators are compounds that promote SIRT1 deacetylase activity [33] in vitro. In vivo, SIRT1 activators prevent RGC loss during EAE optic neuritis [23-25], but specific increase in SIRT1 activity in optic nerve was not assessed. To determine the timing of SIRT1 activity changes in optic nerve, wild-type mice were treated with SIRT1 activator SRTAW04 by oral gavage at a dose of 100 mg/kg/day for 4 days and mice were killed on the 4th day at different time intervals after the final dose. Optic nerves were isolated and SIRT1 activity was determined with a SIRT1 fluorometric substrate assay kit. Results show a significant increase in SIRT1 activity 1 hr after SRTAW04 treatment (Figure 2a). Increased activity was transient, and declined back to control levels after 2 hr.

We next examined SIRT1 activity in the optic nerves of MHV-A59 infected mice after 30 days of SRTAW04 treatment. 4 week old mice were infected with MHV-A59 and were treated with SRTAW04 starting from day 1 with 100 mg/kg/day for 30 days. On the 30th day mice were sacrificed 1 hr after SRTAW04 treatment and protein was extracted from optic nerves for SIRT1 activity assay. Optic nerves of MHV-A59 mice treated with SRTAW04 showed a significant increase in SIRT1 activity compared to control and untreated MHV-A59 infected mice (Figure 2b). Interestingly, untreated MHV-A59 infected mouse optic nerves also showed a smaller but significant increase compared to control, possibly as a natural defense mechanism. We also examined levels of SIRT1 in retinas and optic nerves of mice after 7 or 30 days with or without treatment by SRTAW04. SIRT1 protein expression levels measured by Western blotting showed no significant differences between any treatment groups in day 30 optic nerves (Figure 2c), with similar lack of change in day 7 optic nerves and in retinas at either time point (data not shown).

### SRTAW04 treatment prevents neuronal loss in MHV-A59 infected mice

**Figure 3:**
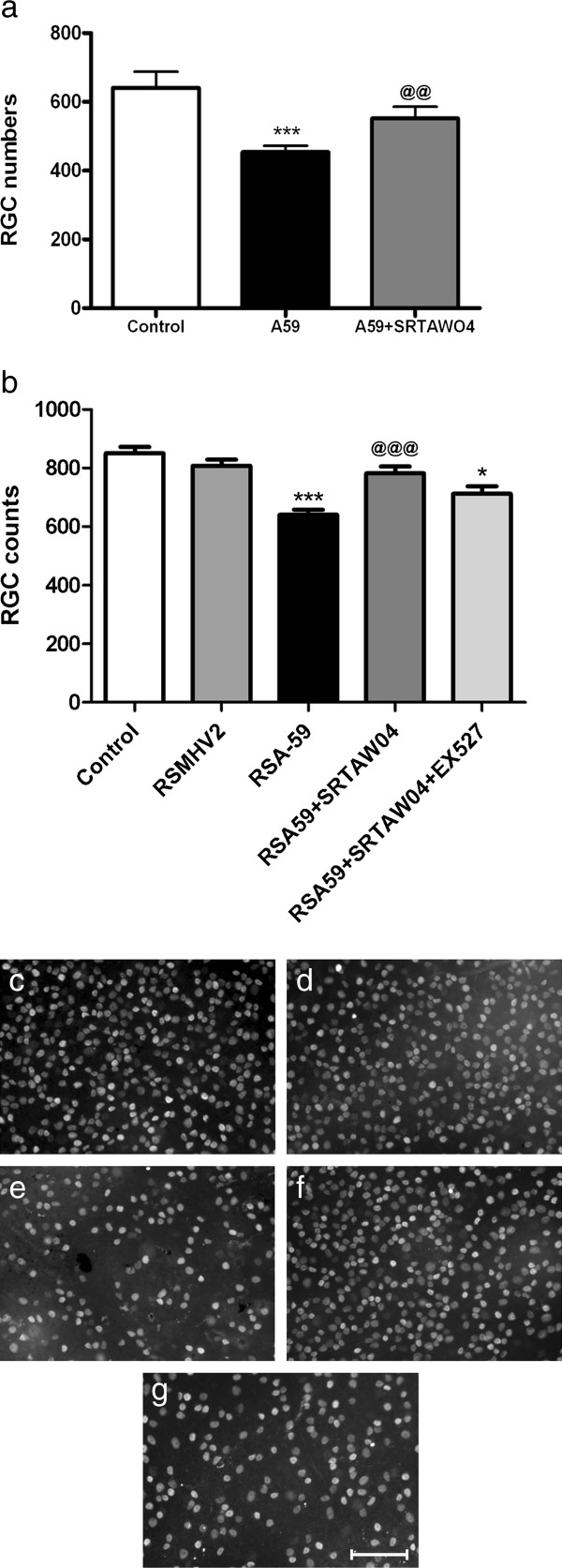
**SRTAW04 treatment prevents neuronal loss in MHV-A59 infected mice. (a)** MHV-A59 (n = 28) infection significantly decreases RGC numbers compared to non-infected control mice (n = 18) (***p < 0.001). SRTAW04 treatment (100 mg/kg/day) for 30 days (n = 16) results in significant (^**@@**^p < 0.01) attenuation of RGC loss. **(b)** C57BL/6 mice were inoculated with recombinant strain of MHV, RSA59 and the treatment group was administered SRTAW04 (100 mg/kg/day) for 30 days with and without SIRT1 inhibitor EX527 (10 mg/kg/day i.p.). After 30 days the RGCs were labeled by Brn3a staining and counted. RSA59 (n = 12) induced a significant decrease in RGCs (***p < 0.001) compared to control (n = 8) and MHV2 infected (n = 6) mice. Treatment with SRTAW04 (n = 10) significantly (^**@@@**^p < 0.001) attenuated RGC loss. The SRTAW04 treatment group receiving EX527 (n = 10) showed a significant decrease (*p < 0.05) in RGC numbers compared to the group that only received SRTAW04 demonstrating that the RGC protective effect of SRTAW04 is dependent on SIRT1. Brn3a labeled RGCs from a representative retinal field in **(c)** control, **(d)** RSMHV2 infected, **(e)**RSA59 infected, **(f)** RSA59 infected, with SRTAW04 treatment and **(g)** RSA59 infected, with SRTAW04 + EX527 treatment. Fewer RGCs are seen in a corresponding area of retina in an MHV-A59 infected mouse. Retina from an MHV-A59 infected mouse treated with SRTAW04 (100 mg/kg/day) for 30 days shows numerous RGCs similar to the non-infected control whereas MHV-A59 infected mouse treated with SRTAW04+ EX527 for 30 days shows fewer RGCs. *Scale bars* 10 μm for **c-g**.

We have shown that SIRT1 activators attenuate RGC loss during EAE optic neuritis [23-25] however, neuronal damage in the MHV model of MS occurs by different mechanisms than in EAE, including direct viral infection of neurons and macrophage-mediated myelin stripping of axons [18]. The ability of SRTAW04 to attenuate neuronal loss in MHV-A59 infected mice was therefore examined. RGCs of 4 week old C57BL/6 mice were labeled with fluorogold and mice were inoculated with MHV-A59 one week later. The treatment group was administered SRTAW04 (100 mg/kg/day) by oral gavage starting from day 1 for 30 days. The decrease in RGC numbers in untreated MHV-A59 mice, compared to controls, was significantly attenuated by SRTAW04 treatment (Figure 3a). To further confirm the neuroprotective effect of SRTAW04, 4 week old C57BL/6 mice were inoculated with recombinant strain of MHV, RSA59 and the treatment group was administered the same dose of SRTAW04 for 30 days with and without SIRT1 inhibitor EX527 (10 mg/kg/day i.p.). After 30 days RGCs were labeled by Brn3a staining and counted. Consistent with MHV-A59 results (Figure 3a) RSA59 induced a significant decrease in RGCs and treatment with SRTAW04 attenuated RGC loss. The SRTAW04 treatment group receiving EX527 showed a significant decrease in RGC numbers compared to the group that only received SRTAW04 (Figure 3b, c) demonstrating that the RGC protective effect of SRTAW04 is dependent on SIRT1. Treatment with SIRT1 inhibitor alone does not further reduce RGC survival (data not shown), similar to prior studies [24,38].

### Effects of SRTAW04 on neuronal loss in the optic nerve

**Figure 4:**
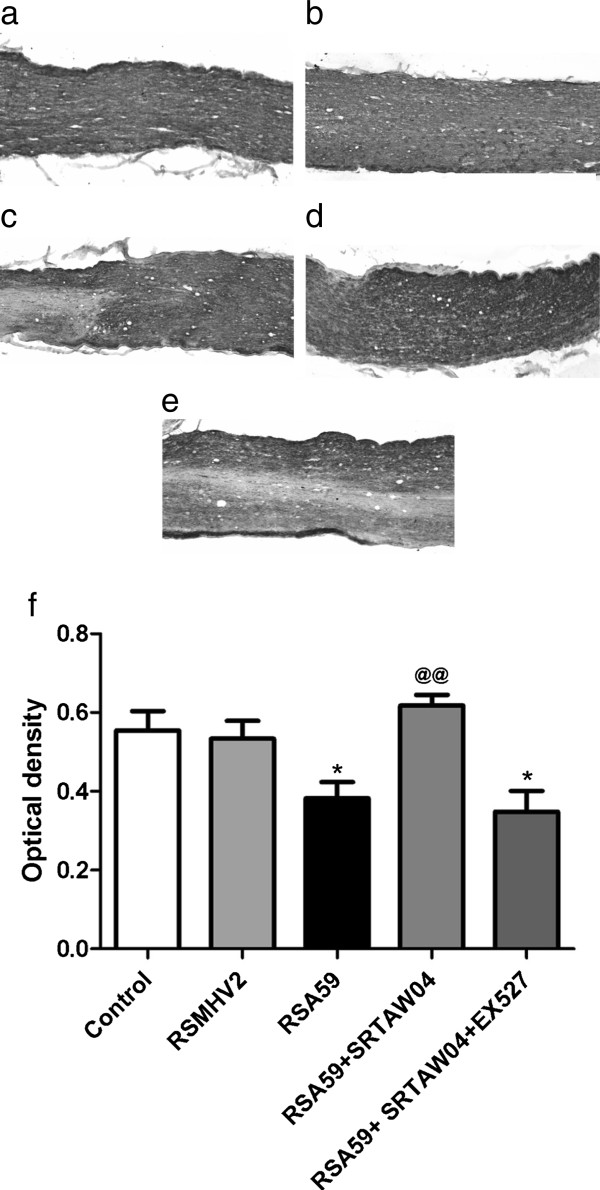
**Effects of SRTAW04 on neuronal loss in the optic nerve.** Neurofilament stained optic nerves from a representative **(a)** control, (**b**) RSMHV2 infected, **(c) **RSA59 infected, **(d)** RSA59 infected, with SRTAW04 treatment and **(e)** RSA59 infected, with SRTAW04 + EX527 treatment demonstrate normal axonal staining **(a,b,d)** and areas of axonal loss **(c,e)**. *Scale bars* 100 μm for **a-e**. **(f)** Neurofilament staining of the optic nerve 30 days postinoculation demonstrates significant loss (*p < 0.05) of axons in mice infected with RSA59 (n = 26) compared to normal axonal staining in control (n = 12) and RSMHV2-infected (n = 10) mice. SRTAW04 treatment (n = 16) shows a significant preservation (^**@@**^p < 0.01) of the axons compared to RSA59 infected mice. SRTAW04 treatment group with EX527 co-treatment (n = 22) shows significant reduction (*p < 0.05) in axonal staining compared to the RSA59 with SRTAW04 treatment group.

To examine whether viral induced ON also leads to axonal loss, optic nerves were stained with anti-neurofilament antibodies and the area of axonal staining was quantified as described previously [18]. Thirty days post inoculation, nerves from RSA59-infected mice showed significantly decreased axonal staining compared to control nerves or nerves from MHV2-infected mice (Figure 4a-c,f). SRTAW04 treatment showed a significant preservation of the axons whereas SRTAW04 treated mice that received EX527 co-treatment showed no change compared to the RSA59 infected group (Figure 4c-e,f).

### SRTAW04 treatment reduces accumulation of ROS in MHV-A59 infected optic nerves

**Figure 5:**
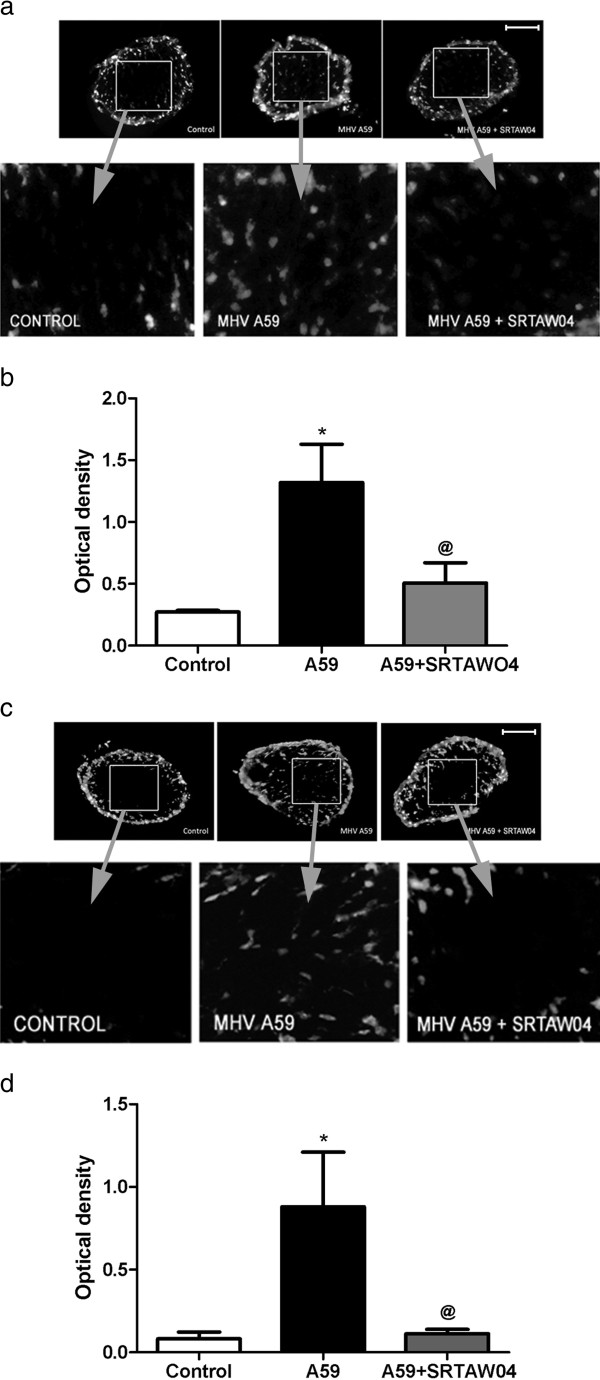
**SRTAW04 treatment reduces ROS in MHV-A59 infected optic nerves both 7 and 30 days after infection.** Mice were infected with MHV-A59 and sacrificed 7 days post-inoculation, when optic nerve inflammation is known to peak. **(a)** Representative images show optic nerves stained with MitoSOX Red. There is an increase in staining of the superoxide anion in MHV-A59 infected optic nerve compared to control optic nerves, with less staining present in optic nerve from an MHV-A59 infected mouse treated with SRTAW04 (100 mg/kg/day) for 7 days. **(b)** Quantification of MitoSOX Red staining in optic nerves. Average optical density in the central area of optic nerves was determined with image J software. Treatment with SRTAW04 (n = 8) significantly (^**@**^p < 0.05) attenuated the increase in superoxide anion staining induced during MHV-A59 (n = 6) infection relative to controls (*p < 0.05). **(c)** Representative images show optic nerves stained with MitoSOX Red 30 days after infection with MHV-A59. There is increased staining in MHV-A59 infected optic nerve compared to control optic nerve, with less staining present in optic nerve from an MHV-A59 infected mouse treated with SRTAW04 (100 mg/kg/day) for 30 days. **(d)** Treatment with SRTAW04 (n = 8) significantly (^**@**^p < 0.05) attenuated the increase in average optical density of superoxide anion staining observed in MHV-A59 (n = 7) infection relative to controls (n = 5) (*p < 0.05). *Scale bars* 100 μm for **a,c**.

MitoSOX Red detection of superoxide within mitochondria was used to determine whether there is ROS accumulation in MHV-A59 induced optic neuritis. Mice were infected with MHV-A59 and sacrificed 7 days post-inoculation, when optic nerve inflammation is known to peak [21]. Optic nerves were isolated and stained with MitoSOX Red, which revealed an increase in the superoxide anion in MHV-A59 infected optic nerve compared to control optic nerves, and optic nerves from MHV-A59 infected mice treated daily with 100 mg/kg SRTAW04 had significantly less staining than untreated MHV-A59 infected mice (Figure 5a, b). We then examined accumulation of superoxide in optic nerves 30 days post-inoculation with MHV-A59 and with or without daily treatment with 100 mg/kg SRTAW04. Results again showed a significant increase in MitoSOX Red staining in optic nerves of mice infected with MHV-A59, as compared to control optic nerves, and treatment with SRTAW04 for 30 days significantly attenuated the accumulation of superoxide staining (Figure 5c, d).

### Effects of SRTAW04 on expression of markers of mitochondrial and anti-oxidant function

**Figure 6:**
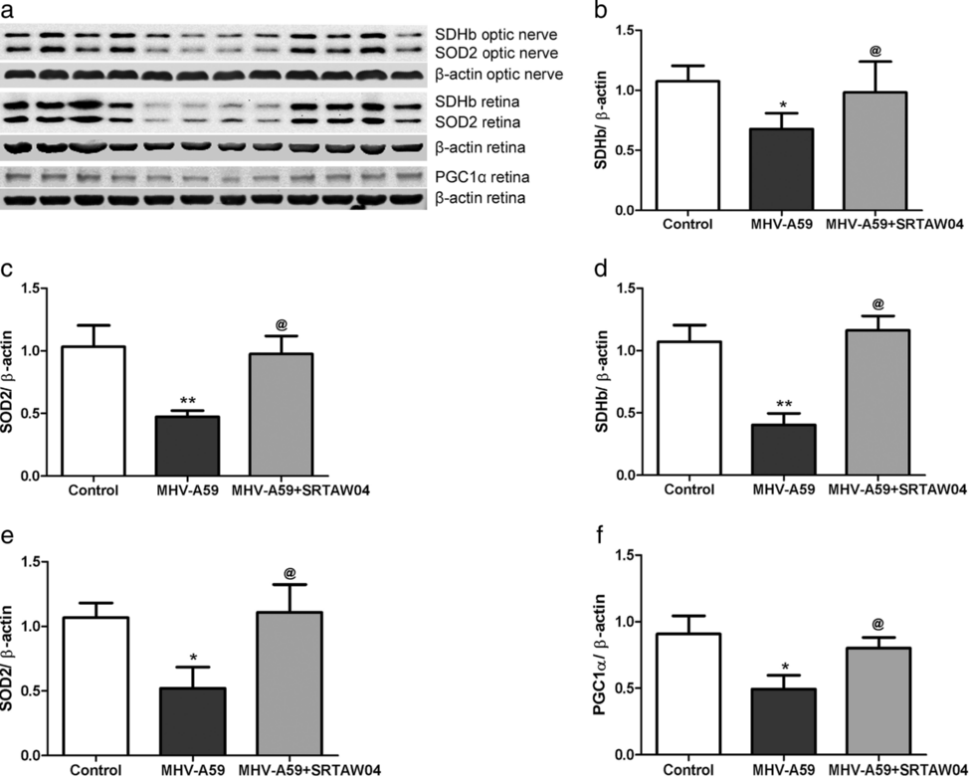
**Effects of SRTAW04 on expression of markers of mitochondrial and anti-oxidant function. (a)** Western blot of protein extracts from optic nerve and retina of control (lanes 1–4), MHV-A59 infected (lanes 5–8), and MHV-A59 infected + SRTAW04-treated (lanes 9–12) mice. Average levels of SDHb measured by Western blotting (n = 4/group) showed a significant (*p < 0.05) decrease in protein extracts from optic nerves **(a,b)** and retinas **(a,d)** of MHV-A59 infected mice 7 days post-inoculation, compared to control mice. MHV-A59 infected mice treated with SRTAW04 (100 mg/kg/day) showed a significant increase (^**@**^p < 0.05) of SDHb protein expression compared to untreated MHV-A59 infected mice. There is a significant decrease (*p < 0.05) in expression of SOD2 (n = 4/group) in optic nerves **(a,c)** and retinas **(a,e)** of MHV-A59 infected mice compared to control mice, and treatment with SRTAW04 significantly (^**@**^p < 0.05) attenuates that change. PGC1-α expression shows a significant (*p < 0.05) decrease in retinas **(a,f)** (n = 4/group) during MHV-A59 infection and treatment with SRTAW04 for 7 days significantly (^**@**^p < 0.05) increases the PGC1-α protein levels.

Optic nerves and retinas were isolated during the peak of inflammation, *7* days post-inoculation with MHV-A59, from mice treated with or without 100 mg/kg SRTAW04 daily, and expression levels of mitochondrial and anti-oxidant markers were measured. SDH is a key mitochondrial enzyme that catalyses oxidation of succinate to fumarate in the Krebs cycle, and feeds electrons to the respiratory chain ubiquinone (UQ) pool [43]. SDH functions not only in mitochondrial energy generation, but also has a role in oxygen sensing [44]. Protein levels of SDHb showed a significant decrease in the MHV-A59 infected group compared to controls. Treatment with SRTAW04 for seven days showed a significant increase of SDHb protein expression when compared to untreated MHV-A59 infected mice in both optic nerve and retina (Figure 6a,b,d). We also measured protein expression of SOD2, a mitochondrial protein which binds to superoxide byproducts of oxidative phosphorylation and converts them to hydrogen peroxide and diatomic oxygen [45]. Results showed a significant decrease in expression of SOD2 in optic nerves and retinas of MHV-A59 infected mice and treatment with SRTAW04 significantly attenuated that change (Figure 6a,c,e). The peroxisome proliferator activated receptor (PPAR) co-activator 1-α (PGC1-α) is a transcriptional co-activator identified as an upstream regulator of mitochondrial number and function [46] and is activated by SIRT1-mediated deacetylation [26]. Results showed a significant decrease in PGC1-α expression during MHV-A59 infection, and treatment with SRTAW04 significantly increases the protein levels in retinas of 7 day treated mice (Figure 6a,f). PGC1-α levels in optic nerve were too low to be detected.

### Effects of SRTAW04 on demyelination in the spinal cord

**Figure 7:**
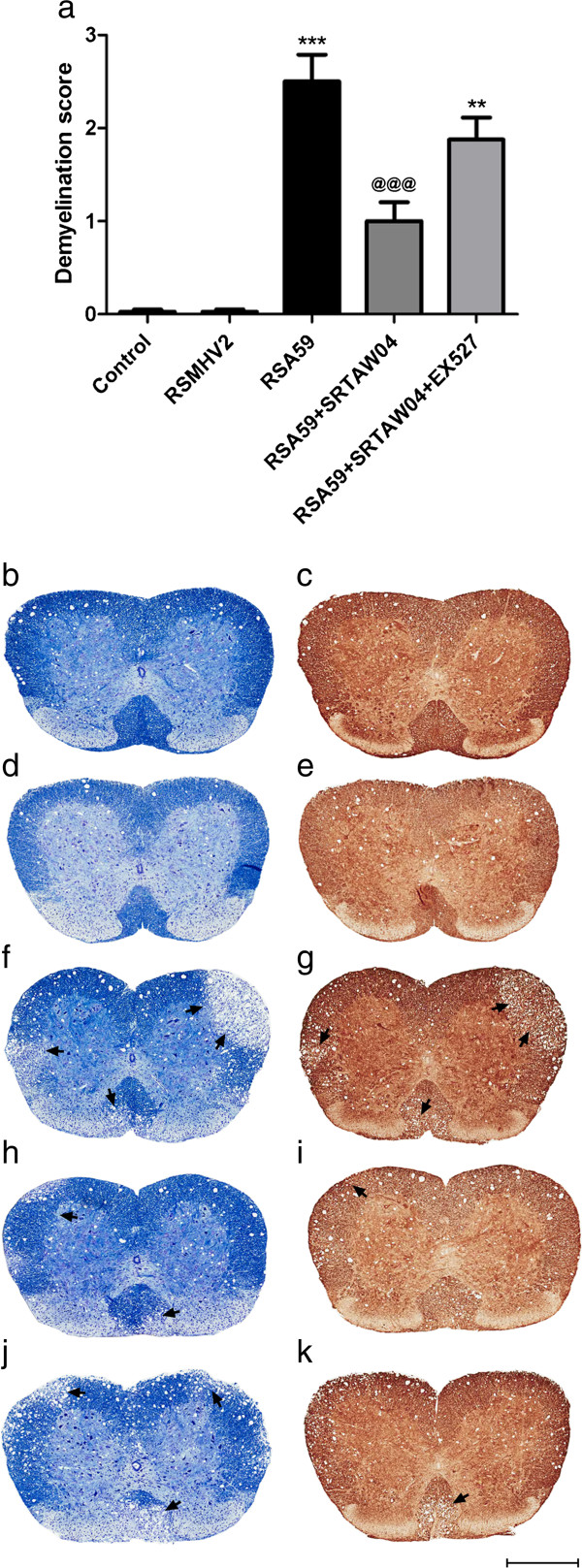
**Effects of SRTAW04 on demyelination in the spinal cord. (a)** Demyelination, scored on a relative scale by histologic evaluation of LFB stained spinal cords (n = 4-5 per group) taken 30 days after infection, shows a significant increase (***p < 0.001) in the RSA59 infected group compared to control and RSMHV2 infected mice. Treatment with SRTAW04 shows a significant reduction in the demyelinating score (^**@@@**^p < 0.001). Treatment group that received SRTAW04 with EX527 showed a significant increase (^**@@**^p < 0.01) in demyelinating score compared to the group that received SRTAW04 alone, but no significant change compared to untreated RSA59 infected mice. LFB (left) and neurofilament (right) stained serial spinal cord sections from a representative **(b,c)** control, **(d,e)** RSMHV2 infected, **(f,g)** RSA59 infected, **(h,i)** RSA59 infected, with SRTAW04 treatment and **(j,k)** RSA59 infected, with SRTAW04 + EX527 treatment mouse. Neurofilament staining shows axonal loss limited to the areas of demyelination seen on LFB stained sections. *Scale bars* 500 μm for **b-k**.

In addition to optic nerve, MHV-A59 and RSA59 spread to spinal cord and induce significant demyelination and axonal injury after intracranial inoculation [18]. We therefore used spinal cord to confirm neuroprotective effects of SRTAW04 in RSA59 infected mice. Since tissue is limited in optic nerve, histologic studies were also performed using spinal cord, which has larger areas of white matter, to assess effects of SRTAW04 on demyelination. Spinal cords were isolated from mice infected with RSA59 for 30 days, treated with or without 100 mg/kg SRTAW04 daily, and demyelination levels were measured using LFB staining. Pathology was assessed in five to seven cross-sections of spinal cord from cervical, thoracic, and lumbar regions. Demyelinating plaques were quantified on a 0 to 3 scale in four quadrants from two spinal cord levels for each mouse. Spinal cord sections from the RSA59 infected group showed a significant increase in the demyelinating score compared to control and RSMHV2 infected groups (Figure 7a,b,d,f). The group receiving SRTAW04 showed a significant decrease in the demyelinating score compared to RSA59 infected group (Figure 7a,f, h). In contrast, SRTAW04 treated mice that also received the SIRT1 inhibitor EX527 did not show a significant reduction in demyelination (Figure 7a, f-j). Serial sections were stained with antibodies for neurofilament and show focal axonal degeneration only in the areas of demyelination. (Figure 7c,e,g,i,k)

### Effects of SRTAW04 on inflammation in the spinal cord

**Figure 8:**
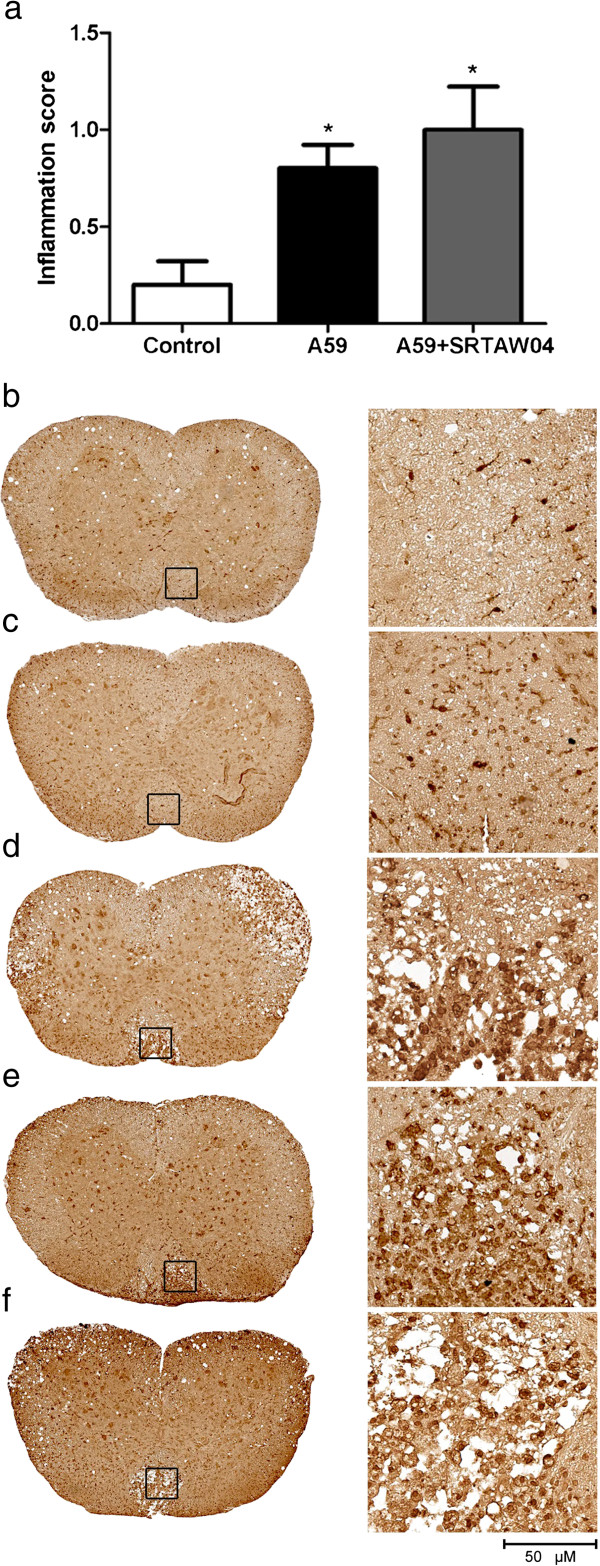
**Effects of SRTAW04 on inflammation in the spinal cord. (a)** Spinal cord sections (n = 5 per group) from 7 day post infected MHV-A59 and MHV-A59 infected group treated with 100 mg/kg SRTAW04 daily stained with H&E were scored using a relative inflammation scale. There is a significant increase (*p < 0.05) in the inflammation score in the MHV-A59 treated mice compared to controls. No significant difference in the inflammation score is observed between the MHV-A59 infected group and MHV-A59 infected mice treated with SRTAW04. By day 30 post-innoculation, limited inflammation is observed by H&E staining in spinal cord, and inflammation that does occur consists almost exclusively of macrophages/microglia, thus sections (n = 4-5 per group) were immunostained for Iba1 to further assess relative levels of inflammation. A representative spinal cord section is shown from **(b)** control, **(c)** RSMHV2 infected, **(d)** RSA59 infected, **(e)** RSA59 infected + SRTAW04 treatment and **(f)** RSA59 infected + SRTAW04 + EX527 treatment mice. Boxes in low magnification sections (left column) indicate area shown at high magnification (right column). Increased foci of Iba1 positive cells are present in RSA59 infected mice with or without SRTAW04 treatment compared to control and RSMHV2 mice. *Scale bars* 50 μm for **b-f**.

To investigate whether SRTAW04 has an effect on inflammation, spinal cords from 7 day p.i. mice with MHV-A59 with or without treatment with 100 mg/kg SRTAW04 daily were cut into 5 μm coronal sections and stained with H&E and scored using a 3 point scale as described previously [21]. There was a significant increase in inflammation in MHV-A59 infected mice compared to controls (Figure 8a). Interestingly there was no significant difference observed between the inflammation score of the MHV-A59 infected group and the MHV-A59 infected mice treated with SRTAW04 (Figure 8a). No significant effect of SRTAW04 on inflammation was observed in H&E stained sections of spinal cord from mice 30 days p.i. although inflammation levels are low (data not shown). To further investigate the effect of SRTAW04 on inflammation 30 days post infection, sections were stained with anti-Iba1 antibody, a macrophage/microglial marker. Results suggest there is difference in the area and number of microglia present, although all plaques contain Iba-1 stained cells regardless of SRTAW04 treatment (Figure 8b-f).

## Discussion

Results confirm that infection with demylinating strains MHV-A59 and RSA59 induce optic neuritis in this model of MS, and for the first time demonstrate significant RGC loss occurs in this model as well. SIRT1 activating compound SRTAW04 significantly attenuates neuronal loss induced by MHV-A59 and RSA59 infection. Similar neuroprotective effects mediated by several SIRT1 activators were previously demonstrated in two different EAE models of MS, relapsing/remitting EAE in SJL/J mice [23,24] and chronic EAE in C57BL/6 mice [22], but the mechanism of these effects was not assessed beyond the role of activating SIRT1. The current results suggest SIRT1 activators can work by promoting mitochondrial function and reducing the accumulation of ROS. While such oxidative stress has been demonstrated previously in EAE [28,30], it was not clear whether similar mechanisms would be found in the MHV induced demyelinating disease. While we found that SIRT1 activation is associated with reduced accumulation of ROS in optic nerves, one limitation of this study that will be addressed in future studies is that a causal relation of this effect has not been confirmed using ROS inhibitors as positive controls.

Interestingly, pathogenesis of neuronal injury begins with different triggers in EAE optic neuritis as compared to MHV optic neuritis, despite similar gross levels of inflammation. Axonal injury and loss of neurons in relapsing/remitting EAE occurs secondary to inflammation [47,48], predominantly mediated by effector T cells, whereas neurotropic strains of MHV can directly infect neurons leading to direct injury, and leading to myelin stripping by activated microglia/macrophages [18]. MHV induced disease also occurs even in the absence of lymphocytes [49]. The ability of SIRT1 activators to prevent neuronal loss in both models therefore suggests they modulate a common mechanism of neurodegeneration downstream of the initial mechanisms of injury. SIRT1 activators appear to work by increasing expression of proteins involved in mitochondrial biogenesis and reduction of ROS, and indicate these are important targets for neuroprotective therapies. Consistent with this, SIRT1 activators were found to have similar neuroprotective effects in a model of traumatic optic nerve injury [38].

Resveratrol, a naturally-occurring polyphenolic compound, is the most studied SIRT1 activator [50]. A role for resveratrol and SIRT1 in neuroprotection has been suggested in other neurodegenerative processes besides demyelinating disease, including models of Alzheimer’s disease [51,52], amyotrophic lateral sclerosis [52], and axotomized dorsal root ganglion cells [50]. However, resveratrol also modulates a variety of cellular signaling pathways independent of its ability to activate SIRT1, exerting a number of potentially beneficial effects including anti-carcinogenic properties, anti-inflammatory effects by inhibiting pro-inflammatory mediators and/or activated immune cells, and inhibition of inducible nitric oxide synthase and cycooxygenase-2 [53,54]. For the current studies, we used the SIRT1 activator SRTAW04 to avoid activating other pathways affected by resveratrol. In addition, compounds like SRTAW04 that activate SIRT1 by distinct mechanisms from resveratrol exert similar SIRT1 activation levels at concentrations an order of magnitude lower than resveratrol [33], and have shown good safety profiles in early phase clinical trials. In addition, we have shown previously that SRTAW04 itself prevents oxidative stress-induced loss of neurons in vitro [27].

Specific targets of the SIRT1 deacetylase that mediate observed changes in expression of mitochondrial enzymes require further study. SIRT1 was originally identified as a histone deacetylase [55,56], leading to large changes in overall gene expression, but SIRT1 also has been found to deacetylate a number of other protein targets [57,58], including transcription factors that may directly affect expression of specific genes. In addition, post-translational modifications of mitochondrial enzymes may further promote mitochondrial biogenesis, as SIRT1-mediated deacetylation of PGC1-α has been shown to do in muscle cells [26]. Results here show that transient increases in SIRT1 activity following administration of SRTAW04 were capable of providing long-term neuroprotective effects in MHV-A59 infected mice, suggesting that deacetylation of SIRT1 substrates may persist beyond the timing of acute SIRT1 activation.

The current studies show SRTAW04 exerts neuroprotective effects without suppressing gross levels of inflammation assessed by H&E staining, and confirmed by staining with macrophage/microglial markers. Unlike EAE models of MS mediated by T cells, in this virus induced model of MS inflammation consists almost exclusively of macrophages, as demonstrated in prior studies [18,21,42]. It is interesting that SIRT1 activation fails to block the migration of macrophages into the CNS, as prior studies have shown that SIRT1 activation can block the accumulation of macrophages in peripheral tissues [59], although even outside the CNS this effect appears to be tissue specific.

Evidence suggests neurologic dysfunction in MS occurs as a result of axonal degeneration [60,61], which may be dependent on or occur independently of chronic demyelination [62]. Our results show that SIRT1 activation can preserve RGCs and reduce axonal loss following infection with MHV. SRTAW04 treated mice that also received the SIRT1 inhibitor EX527 did not show a significant reduction in axonal loss or preserve RGCs, suggesting that the neuroprotective effect of SRTAW04 is mediated by SIRT1 activation. Furthermore, SIRT1 activation with SRTAW04 prevented myelin loss following MHV infection. These findings are consistent with a recent study [63] showing that mice overexpressing SIRT1 in neurons have reduced axonal loss and demyelination in the EAE model of MS. Together, results suggest SIRT1 promotes neuronal survival and prevents demyelination in CNS demyelinating diseases.

## Conclusions

SIRT1 activating compounds represent potential new neuroprotective agents for demyelinating diseases. Results suggest SIRT1 activators can modulate oxidative stress, a common mechanism of neuronal injury, and begin to identify mechanisms that can be targeted for development of more specific neuroprotective therapies.

## Competing interests

SRTAW04 was provided at no cost by Sirtris, a GSK Company, to KSS for these studies. No funding was provided by Sirtris for the studies, and KSS has no financial interests or relationships with Sirtris or related to its SRTAW04 compound. The authors have no other competing interests to declare.

## Authors’ contributions

RSK helped design these studies, performed all experiments, analyzed the data, and prepared the manuscript. KD assisted with induction of MHV disease, prepared tissue sections, and performed histological studies. JDS helped with experimental design, data analysis and preparation of the manuscript. KSS oversaw all aspects of these studies and was involved in experimental design, data analysis and manuscript preparation. All authors read and approved the final manuscript.

## References

[B1] Noseworthy JH Lucchinetti C Rodriguez M Weinshenker BG Multiple sclerosis N Engl J Med 2000 2 938 952 10.1056/NEJM200009283431307 11006371

[B2] Davie CA Barker GJ Webb S Tofts PS Thompson AJ Harding AE McDonald WI Miller DH Persistent functional deficit in multiple sclerosis and autosomal dominant cerebellar ataxia is associated with axon loss Brain 1995 2 1583 1592 10.1093/brain/118.6.1583 8595487

[B3] Losseff NA Webb SL O'Riordan JI Page R Wang L Barker GJ Tofts PS McDonald WI Miller DH Thompson AJ Spinal cord atrophy and disability in multiple sclerosis. A new reproducible and sensitive MRI method with potential to monitor disease progression Brain 1996 2 701 708 10.1093/brain/119.3.701 8673483

[B4] Losseff NA Wang L Lai HM Yoo DS Gawne-Cain ML McDonald WI Miller DH Thompson AJ Progressive cerebral atrophy in multiple sclerosis. A serial MRI study Brain 1996 2 2009 2019 10.1093/brain/119.6.2009 9010005

[B5] Trip SA Schlottmann PG Jones SJ Altmann DR Garway-Heath DF Thompson AJ Plant GT Miller DH Retinal nerve fiber layer axonal loss and visual dysfunction in optic neuritis Ann Neurol 2005 2 383 391 10.1002/ana.20575 16075460

[B6] Fisher JB Jacobs DA Markowitz CE Galetta SL Volpe NJ Nano-Schiavi ML Baier ML Frohman EM Winslow H Frohman TC Calabresi PA Maguire MG Cutter GR Balcer LJ Relation of visual function to retinal nerve fiber layer thickness in multiple sclerosis Ophthalmology 2006 2 324 332 10.1016/j.ophtha.2005.10.040 16406539

[B7] Johnson KP Brooks BR Cohen JA Ford CC Goldstein J Lisak RP Myers LW Panitch HS Rose JW Schiffer RB Vollmer T Weiner LP Wolinsky JS Copolymer 1 reduces relapse rate and improves disability in relapsing-remitting multiple sclerosis: results of a phase III multicenter, double-blind, placebo-controlled trial Neurology 2001 2 S16 S24 11902590

[B8] Jacobs LD Cookfair DL Rudick RA Herndon RM Richert JR Salazar AM Fischer JS Goodkin DE Granger CV Simon JH Alam JJ Bartoszak DM Bourdette DN Braiman J Brownscheidle CM Coats ME Cohan SL Dougherty DS Kinkel RP Mass MK Intramuscular interferon beta-1a for disease progression in relapsing multiple sclerosis Ann Neurol 1996 2 285 294 10.1002/ana.410390304 8602746

[B9] Parry A Corkill R Blamire AM Palace J Narayanan S Arnold D Styles P Matthews PM Beta-Interferon treatment does not always slow the progression of axonal injury in multiple sclerosis J Neurol 2003 2 171 178 10.1007/s00415-003-0965-8 12574947

[B10] Hickman SJ Kapoor R Jones SJ Altmann DR Plant GT Miller DH Corticosteroids do not prevent optic nerve atrophy following optic neuritis J Neurol Neurosurg Psychiatry 2003 2 1139 1141 10.1136/jnnp.74.8.1139 12876255 PMC1738596

[B11] Gilden DH Infectious causes of multiple sclerosis Lancet Neurol 2005 2 195 202 15721830 10.1016/S1474-4422(05)01017-3 PMC7129502

[B12] Constantinescu CS Hilliard B Fujioka T Bhopale MK Calida D Rostami AM Pathogenesis of neuroimmunologic diseases, Experimental models Immunol Res 1998 2 217 227 10.1007/BF02786446 9479583

[B13] Kornek B Storch MK Weissert R Wallstroem E Stefferl A Olsson T Linington C Schmidbauer M Lassmann H Multiple sclerosis and chronic autoimmune encephalomyelitis: a comparative quantitative study of axonal injury in active, inactive, and remyelinated lesions Am J Pathol 2000 2 267 276 10.1016/S0002-9440(10)64537-3 10880396 PMC1850217

[B14] Meyer R Weissert R Diem R Storch MK de Graaf KL Kramer B Bahr M Acute neuronal apoptosis in a rat model of multiple sclerosis J Neurosci 2001 2 6214 6220 11487644 10.1523/JNEUROSCI.21-16-06214.2001 PMC6763179

[B15] Wujek JR Bjartmar C Richer E Ransohoff RM Yu M Tuohy VK Trapp BD Axon loss in the spinal cord determines permanent neurological disability in an animal model of multiple sclerosis J Neuropathol Exp Neurol 2002 2 23 32 11829341 10.1093/jnen/61.1.23

[B16] Lavi E Gilden DH Wroblewska Z Rorke LB Weiss SR Experimental demyelination produced by the A59 strain of mouse hepatitis virus Neurology 1984 2 597 603 10.1212/WNL.34.5.597 6324031

[B17] Lavi E Gilden DH Highkin MK Weiss SR MHV-A59 pathogenesis in mice Adv Exp Med Biol 1984 2 237 245 10.1007/978-1-4615-9373-7_24 6331114

[B18] Das Sarma J Kenyon LC Hingley ST Shindler KS Mechanisms of primary axonal damage in a viral model of multiple sclerosis J Neurosci 2009 2 10272 10280 10.1523/JNEUROSCI.1975-09.2009 19692601 PMC2747667

[B19] Arnold AC Evolving management of optic neuritis and multiple sclerosis Am J Ophthalmol 2005 2 1101 1108 10.1016/j.ajo.2005.01.031 15953446

[B20] Shindler KS Guan Y Ventura E Bennett J Rostami A Retinal ganglion cell loss induced by acute optic neuritis in a relapsing model of multiple sclerosis Mult Scler 2006 2 526 532 10.1177/1352458506070629 17086896

[B21] Shindler KS Kenyon LC Dutt M Hingley ST Das Sarma J Experimental optic neuritis induced by a demyelinating strain of mouse hepatitis virus J Virol 2008 2 8882 8886 10.1128/JVI.00920-08 18579591 PMC2519666

[B22] Quinn TA Dutt M Shindler KS Optic neuritis and retinal ganglion cell loss in a chronic murine model of multiple sclerosis Front Neurol 2011 2 50 21852980 10.3389/fneur.2011.00050 PMC3151613

[B23] Shindler KS Ventura E Rex TS Elliott P Rostami A SIRT1 Activation Confers Neuroprotection in Experimental Optic Neuritis Invest Ophthalmol Vis Sci 2007 2 3602 3609 10.1167/iovs.07-0131 17652729 PMC1964753

[B24] Shindler KS Ventura E Dutt M Elliott P Fitzgerald DC Rostami A Oral resveratrol reduces neuronal damage in a model of multiple sclerosis J Neuroophthalmol 2010 2 328 339 10.1097/WNO.0b013e3181f7f833 21107122 PMC3312784

[B25] Fonseca-Kelly Z Nassrallah M Uribe J Khan RS Dine K Dutt M Shindler KS Resveratrol neuroprotection in a chronic mouse model of multiple sclerosis Front Neurol 2012 2 84 22654783 10.3389/fneur.2012.00084 PMC3359579

[B26] Lagouge M Argmann C Gerhart-Hines Z Meziane H Lerin C Daussin F Messadeq N Milne J Lambert P Elliott P Geny B Laakso M Puigserver P Auwerx J Resveratrol improves mitochondrial function and protects against metabolic disease by activating SIRT1 and PGC-1alpha Cell 2006 2 1109 1122 10.1016/j.cell.2006.11.013 17112576

[B27] Khan RS Fonseca-Kelly Z Callinan C Zuo L Sachdeva MM Shindler KS SIRT1 activating compounds reduce oxidative stress and prevent cell death in neuronal cells Front Cell Neurosci 2012 2 1 13 23293585 10.3389/fncel.2012.00063 PMC3533205

[B28] Guy J Ellis EA Mames R Rao NA Role of hydrogen peroxide in experimental optic neuritis. A serial quantitative ultrastructural study Ophthalmic Res 1993 2 253 264 10.1159/000267321 8233351

[B29] Dutta R McDonough J Yin X Peterson J Chang A Torres T Gudz T Macklin WB Lewis DA Fox RJ Rudick R Mirnics K Trapp BD Mitochondrial dysfunction as a cause of axonal degeneration in multiple sclerosis patients Ann Neurol 2006 2 478 489 10.1002/ana.20736 16392116

[B30] Qi X Lewin AS Sun L Hauswirth WW Guy J Suppression of mitochondrial oxidative stress provides long-term neuroprotection in experimental optic neuritis Invest Ophthalmol Vis Sci 2007 2 681 691 10.1167/iovs.06-0553 17251466

[B31] Das Sarma J Fu L Weiss SR Lavi E Demyelination determinants in the S gene of MHV Adv Exp Med Biol 2001 2 133 137 10.1007/978-1-4615-1325-4_21 11774457

[B32] Das Sarma J Iacono K Gard L Marek R Kenyon LC Koval M Weiss SR Demyelinating and nondemyelinating strains of mouse hepatitis virus differ in their neural cell tropism J Virol 2008 2 5519 5526 10.1128/JVI.01488-07 18385249 PMC2395180

[B33] Milne JC Lambert PD Schenk S Carney DP Smith JJ Gagne DJ Jin L Boss O Perni RB Vu CB Bemis JE Xie R Disch JS Ng PY Nunes JJ Lynch AV Yang H Galonek H Israelian K Choy W Small molecule activators of SIRT1 as therapeutics for the treatment of type 2 diabetes Nature 2007 2 712 716 10.1038/nature06261 18046409 PMC2753457

[B34] Cohen-Kfir E Artsi H Bajayo A Gabet Y Gurt I Kalish N Shahar R Dresner-Pollak R The Sirtuin 1 Activator SRTAW04 restores Bone Mass and Structure in Ovariectomized Mice A Potential Novel Therapy for Osteoporosis Endocr Rev 2013 2 OR24 26

[B35] Dietrich MO Antunes C Geliang G Liu ZW Borok E Nie Y Xu AW Souza DO Gao Q Diano S Gao XB Horvath TL Agrp neurons mediate Sirt1’s action on the melanocortin system and energy balance: roles for Sirt1 in neuronal firing and synaptic plasticity J Neurosci 2010 2 11815 11825 10.1523/JNEUROSCI.2234-10.2010 20810901 PMC2965459

[B36] Peck B Chen CY Ho KK Di Fruscia P Myatt SS Coombes RC Fuchter MJ Hsiao CD Lam EW SIRT inhibitors induce cell death and p53 acetylation through targeting both SIRT1 and SIRT2 Mol Cancer Ther 2010 2 844 855 20371709 10.1158/1535-7163.MCT-09-0971

[B37] Yamaguchi H Woods NT Piluso LG Lee HH Chen J Bhalla KN Monteiro A Liu X Hung MC Wang HG p53 acetylation is crucial for its transcription-independent proapoptotic functions J Biol Chem 2009 2 11171 83 19265193 10.1074/jbc.M809268200 PMC2670122

[B38] Zuo L Khan RS Lee V Dine K Wu W Shindler KS SIRT1 Promotes RGC Survival and Delays Loss of Function Following Optic Nerve Crush Invest Ophthalmol Vis Sci 2013 2 5097 5102 10.1167/iovs.13-12157 23821198 PMC3726244

[B39] Salinas-Navarro M Mayor-Torroglosa S Jimenez-Lopez M Aviles-Trigueros M Holmes TM Lund RD Villegas-Perez MP Vidal-Sanz M A computerized analysis of the entire retinal ganglion cell population and its spatial distribution in adult rats Vision Res 2009 2 115 126 10.1016/j.visres.2008.09.029 18952118

[B40] Forte M Gold BG Marracci G Chaudhary P Basso E Johnsen D Yu X Fowlkes J Rahder M Stem K Bernardi P Bourdette D Cyclophilin D inactivation protects axons in experimental autoimmune encephalomyelitis an animal model of multiple sclerosis Proc Natl Acad Sci 2007 2 7558 7563 17463082 10.1073/pnas.0702228104 PMC1857227

[B41] Das Sarma J Scheen E Seo SH Koval M Weiss SR Enhanced green fluorescent protein expression may be used to monitor murine coronavirus spread in vitro and in the mouse central nervous system J Neurovirol 2002 2 381 391 10.1080/13550280260422686 12402164 PMC7095158

[B42] Shindler KS Chatterjee D Biswas K Goyal A Dutt D Nassrallah M Khan RS Das Sarma J Macrophage-mediated optic neuritis induced by retrograde axonal transport of spike gene recombinant mouse hepatitis virus J Neuropathol Exp Neurol 2011 2 470 480 10.1097/NEN.0b013e31821da499 21572336 PMC3110774

[B43] Ackrell BA Maguire JJ Dallman PR Kearney EB Effect of iron deficiency on succinate- and NADH-ubiquinone oxidoreductases in skeletal muscle mitochondria J Biol Chem 1984 2 10053 10059 6432778

[B44] Baysal BE A phenotypic perspective on Mammalian oxygen sensor candidates Ann N Y Acad Sci 2006 2 221 233 10.1196/annals.1353.024 17102090

[B45] Suski JM Lebiedzinska M Bonora M Pinton P Duszynski J Wieckowski MR Relation between mitochondrial membrane potential and ROS formation Methods Mol Biol 2012 2 183 205 10.1007/978-1-61779-382-0_12 22057568

[B46] Russell LK Mansfield CM Lehman JJ Kovacs A Courtois M Saffitz JE Cardiac-specific induction of the transcriptional coactivator peroxisome proliferator-activated receptor gamma coactivator-1alpha promotes mitochondrial biogenesis and reversible cardiomyopathy in a developmental stage-dependent manner Circ Res 2004 2 525 533 10.1161/01.RES.0000117088.36577.EB 14726475

[B47] Shindler KS Ventura E Dutt M Rostami AM Inflammatory demyelination induces axonal injury and retinal ganglion cell apoptosis in experimental optic neuritis Exp Eye Res 2008 2 208 213 10.1016/j.exer.2008.05.017 18653182 PMC2564281

[B48] Dutt M Tabuena P Ventura E Rostami AM Shindler KS Timing of corticosteroid therapy is critical to prevent retinal ganglion cell loss in experimental optic neuritis Invest Ophthalmol Vis Sci 2010 2 1439 1445 10.1167/iovs.09-4009 19892867 PMC2868414

[B49] Matthews AE Lavi E Weiss SR Neither B cells nor T cells are required for CNS demyelination in mice persistently infected with MHV-A59 J Neurovirol 2002 2 257 264 10.1080/13550280290049697 12053280 PMC7095043

[B50] de la Lastra CA Villegas I Resveratrol as an anti-inflammatory and anti-aging agent: mechanisms and clinical implications Mol Nutr Food Res 2005 2 405 430 10.1002/mnfr.200500022 15832402

[B51] Parker JA Arango M Abderrahmane S Lambert E Tourette C Catoire H Néri C Resveratrol rescues mutant polyglutamine cytotoxicity in nematode and mammalian neurons Nat Genet 2005 2 349 350 10.1038/ng1534 15793589

[B52] Kim D Nguyen MD Dobbin MM Fischer A Sananbenesi F Rodgers JT Delalle I Baur JA Sui G Armour SM Puigserver P Sinclair DA Tsai LH SIRT1 deacetylase protects against neurodegeneration in models for Alzheimer’s disease and amyotrophic lateral sclerosis EMBO J 2007 2 3169 3179 10.1038/sj.emboj.7601758 17581637 PMC1914106

[B53] Araki T Sasaki Y Millbrandt J Increased nuclear NAD biosynthesis and SIRT1 activation prevent axonal degeneration Science 2004 2 1010 1013 10.1126/science.1098014 15310905

[B54] Jang M Cai L Udeani GO Slowing KV Thomas CF Beecher CW Fong HH Farnsworth NR Kinghorn AD Mehta RG Moon RC Pezzuto JM Cancer chemopreventive activity of resveratrol, a natural product derived from grapes Science 1997 2 218 220 10.1126/science.275.5297.218 8985016

[B55] Imai S Armstrong CM Kaeberlein M Guarente L Transcriptional silencing and longevity protein Sir2 is an NAD-dependent histone deacetylase Nature 2000 2 795 800 10.1038/35001622 10693811

[B56] Landry J Sutton A Tafrov ST Heller RC Stebbins J Pillus L Sternglanz R The silencing protein SIR2 and its homologs are NAD-dependent protein deacetylases Proc Natl Acad Sci U S A 2000 2 5807 5811 10.1073/pnas.110148297 10811920 PMC18515

[B57] Blander G Guarente L The Sir2 family of protein deacetylases Annu Rev Biochem 2004 2 417 435 10.1146/annurev.biochem.73.011303.073651 15189148

[B58] Porcu M Chiarugi A The emerging therapeutic potential of sirtuin-interacting drugs: from cell death to lifespan extension Trends Pharmacol Sci 2005 2 94 103 10.1016/j.tips.2004.12.009 15681027

[B59] Lee SM Yang H Tartar DM Gao B Luo X Ye SQ Zaghouani H Fang D Prevention and treatment of diabetes with resveratrol in a non obese mouse model of type 1 diabetes Diabetologia 2011 2 1136 1146 10.1007/s00125-011-2064-1 21340626 PMC4036531

[B60] Budde MD Kim JH Liang HF Russell JH Cross AH Song SK Axonal injury detected by in vivo diffusion tensor imaging correlates with neurological disability in a mouse model of multiple sclerosis NMR Biomed 2008 2 589 597 10.1002/nbm.1229 18041806 PMC2602834

[B61] Budde MD Xie M Cross AH Song SK Axial diffusivity is the primary correlate of axonal injury in the experimental autoimmune encephalomyelitis spinal cord: a quantitative pixelwise analysis J Neurosci 2009 2 2805 2813 10.1523/JNEUROSCI.4605-08.2009 19261876 PMC2673458

[B62] Dandekar AA Wu GF Pewe L Perlman S Axonal damage is T cell mediated and occurs concomitantly with demyelination in mice infected with a neurotropic coronavirus J Virol 2001 2 6115 6120 10.1128/JVI.75.13.6115-6120.2001 11390613 PMC114327

[B63] Nimmagadda VK Bever CT Vattikunta NR Talat S Ahmad V Nagalla NK Trisler D Judge SI Royal W 3rd Chandrasekaran K Russell JW Makar TK Overexpression of SIRT1 protein in neurons protects against autoimmune encephalomyelitis through activation of multiple SIRT1 targets J Immunol 2013 2 4595 4607 23547115 10.4049/jimmunol.1202584 PMC3963275

